# Accounting for Behavior in Treatment Effects: New Applications for Blind Trials

**DOI:** 10.1371/journal.pone.0127227

**Published:** 2015-06-10

**Authors:** Sylvain Chassang, Erik Snowberg, Ben Seymour, Cayley Bowles

**Affiliations:** 1 Department of Economics and Woodrow Wilson School, Princeton University. Princeton, NJ, USA; 2 Division of the Humanities and Social Sciences, California Institute of Technology. Pasadena, CA, USA; 3 National Bureau of Economic Research. Cambridge, MA, USA; 4 Computational and Biological Learning Laboratory, Department of Engineering, University of Cambridge. Cambridge, UK; 5 Center for Information and Neural Networks, National Institute for Information and Communications Technology. Osaka, JAPAN; 6 School of Public Health, Harvard University. Cambridge, MA, USA

## Abstract

The double-blind randomized controlled trial (DBRCT) is the gold standard of medical research. We show that DBRCTs fail to fully account for the efficacy of treatment if there are interactions between treatment and behavior, for example, if a treatment is more effective when patients change their exercise or diet. Since behavioral or *placebo* effects depend on patients’ beliefs that they are receiving treatment, clinical trials with a single probability of treatment are poorly suited to estimate the additional treatment benefit that arises from such interactions. Here, we propose methods to identify interaction effects, and use those methods in a meta-analysis of data from blinded anti-depressant trials in which participant-level data was available. Out of six eligible studies, which included three for the selective serotonin re-uptake inhibitor paroxetine, and three for the tricyclic imipramine, three studies had a high (>65%) probability of treatment. We found strong evidence that treatment probability affected the behavior of trial participants, specifically the decision to drop out of a trial. In the case of paroxetine, but not imipramine, there was an interaction between treatment and behavioral changes that enhanced the effectiveness of the drug. These data show that standard blind trials can fail to account for the full value added when there are interactions between a treatment and behavior. We therefore suggest that a new trial design, two-by-two blind trials, will better account for treatment efficacy when interaction effects may be important.

## Introduction

The expectation of treatment can change the unconscious and conscious behavior of experimental participants [1–3], and may therefore affect measured treatment effects [4]. For example, a participant who believes he or she is receiving treatment may decide to engage in a number of lifestyle changes. He or she may unconsciously spend less time worrying about their illness, or consciously decide to change diet, start exercising, or even socialize more. Placebo effects include the therapeutic effects of such (unconscious or conscious) behavior changes [5], and negative effects, although possibly less common, are called nocebo effects [6, 7].

Blind trials produce consistent expectations of treatment between treatment and control groups, so that the behavior of these groups is the same in aggregate. This ensures that the effects measured by a blind trial are due to the treatment, rather than purely behavioral or placebo effects. While blind trials successfully parcel out pure behavioral effects, they may also fail to account for the value added of treatment when there are interaction effects between treatment and changes in behavior. We show that this issue can be addressed by randomizing the probability *p* of treatment in the trial.

Consider a hypothetical antidepressant that, unknown to the experimenter, works by controlling social anxiety, allowing for more positive interactions in social situations and corresponding reductions in feelings of depression. Thus, the drug is effective in reducing depression among those that decide to socialize more, but has no effect on participants who do not. The probability *p* of treatment in a blind trial will likely influence the participants’ behavior: participants treated with probability *p* = 50% (1/1 odds) will expect more social anxiety than participants treated with probability *p* = 75% (3/1 odds). As such, those with 1/1 odds of treatment may not socialize at all, while those with 3/1 odds may, on average, be more likely to attend social gatherings. As a result, a blind trial with 1/1 odds of treatment would measure no effect, as the treatment is ineffective without changes in behavior. In contrast, a blind trial with 3/1 odds of treatment would measure an effect, as participants in that trial socialize more.

## Methods

A simple formalization of this example clarifies why standard blind trials are generally not suited to account for a treatment’s value added when there are interaction effects between treatment and behavior, and how better trials can be designed to account for such interaction effects. For simplicity, we index the degree of behavioral change by a number *b*(*p*) ∈ [0, 1] with *b*(*p*) = 0 corresponding to no change, and *b*(*p*) = 1 corresponding to complete change. As argued above, the probability of treatment *p* is an important design parameter which affects the behavior *b*(*p*) of participants. Note that participants need not have a precise understanding of probabilities for this to be true. It just needs to be the case that participants feel that 3/1 odds of treatment are greater than 1/1 odds of treatment.

The health outcome of interest *Y* depends both on a participant’s treatment status *τ* ∈ {0,1}, and on a participant’s behavior *b*(*p*) as follows: for all *τ* ∈ {0,1}, *b*(*p*) ∈ [0, 1],

$$\begin{array}{c} Y_{\tau ,p}=\underset{\text{baseline}\;\text{outcome}}{\underset{︸}{\alpha }}+\underset{\text{effect}\;\text{of}\;\text{treatment}\;\text{alone}}{\underset{︸}{\tau \times E_{T}}}+\underset{\text{effect}\;\text{of}\;\text{behavior}\;\text{alone}}{\underset{︸}{b\left(p\right)\times E_{B}}}+\underset{\text{interaction}\;\text{effects}}{\underset{︸}{\tau \times b\left(p\right)\times E_{I}}}+\underset{\text{error}\;\text{term}}{\underset{︸}{U_{Y}}} \end{array}$$ (1)

where *E*_*T*_ is the effect of treatment alone, *E*_*B*_ is the effect of behavior change alone, *E*_*I*_ is the effect of interactions between treatment and behavior. Both effects *E*_*T*_ and *E*_*I*_ contribute to the value added of the intervention since neither can be obtained without the treatment. In contrast, pure behavioral effects *E*_*B*_ can be obtained without the treatment and do not contribute to the value added. (See Technical Appendix 1 for a general derivation of Eq (1).)

To see why standard blind trials might be suboptimal, consider a design in which participants are treated with probability *p*, say *p* = 0.5. The treatment effect estimated by this blind trial is

$$\begin{array}{c} Y_{1,50\%}-Y_{0,50\%}=E_{T}+b\left(p\;=\;0.5\right)\times E_{I}. \end{array}$$

If there are interaction effects between treatment and behavior (that is, *E*_*I*_ ≠ 0), and if 1/1 odds of treatment are not sufficient for participants to engage in large behavioral changes (that is, *b*(50%) ≪ 0.5), this blind trial does not fully account for the antidepressant’s value added.

Moreover, standard blind trials do not separately identify *E*_*T*_ and *E*_*I*_, which matters for future experimental and treatment decisions. Large interaction effects between treatment and behavior warrants further research to understand precisely what changes in behavior matter for outcomes. This will also be important information to convey to both physicians and participants.

This issue can be overcome by implementing a two-by-two factorialized design, where the first factor is the presence or absence of treatment, and the second is the treatment probability and the corresponding behavioral response. The insight here is that by randomizing the probability with which participants are treated, one obtains exogenous variation in behavior patterns [2, 3]. Two-by-two blind trials proceed in two stages:

1. participants are randomly allocated to two arms: a blind trial with a high probability *p*_*H*_ of being treated, and a blind trial with low probability *p*_*L*_ of being treated;
2. participants are informed of their probability of being treated and the blind trials are run in the usual way.

**Fig 1:**
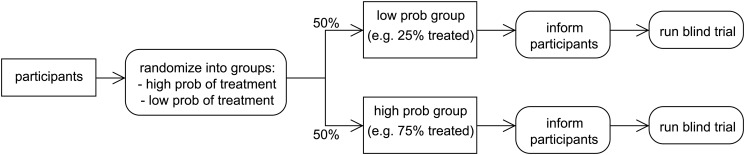
A Two-by-Two Blind Trial.

This is illustrated in Fig 1, which shows the two stages of randomization, with participants first allocated to either a high- or low-probability treatment group, then informed of this probability (thus generating the corresponding placebo effect), and then receiving either treatment or non-treatment in a standard, blinded manner.

The experimenter should make probabilities *p*_*L*_ and *p*_*H*_ sufficiently different that they can realistically induce different behavior patterns. Varying the probability of treatment allows the separate identification of treatment, behavior, and interaction effects as follows:

- $\hat{T}\equiv 𝔼[Y_{1,p_{L}}-Y_{0,p_{L}}]$ is the pure effect of treatment under the default distribution of behavior given low probability of treatment;
- $\hat{B}\equiv 𝔼[Y_{0,p_{H}}-Y_{0,p_{L}}]$ is the pure effect of change in behavior due to greater anticipation of treatment, conditional on no treatment;
- $\hat{I}\equiv 𝔼[Y_{1,p_{H}}-Y_{0,p_{H}}]-𝔼[Y_{1,p_{L}}-Y_{0,p_{L}}]$ is the interaction effect between treatment and changes in behavior, that is, the differential effect of getting treatment between participants in the low probability of treatment and participants in the high probability of treatment groups.

In the previous example, assuming that *b*(*p*_*L*_) ≃ 0 and *b*(*p*_*H*_) ≃ 1, we fully identify the underlying parameters of interest: $\hat{T}=E_{T}$, $\hat{B}=E_{B}$ and $\hat{I}=E_{I}$. More importantly, the interpretation of estimators $\hat{T}$, $\hat{B}$ and $\hat{I}$ as treatment, behavior, and interaction effects, does not rely on the idealized setting of our example, but holds in all generality. For instance, we can allow for arbitrarily complex and random behavioral patterns, as well as heterogeneity in participants’ behavior, beliefs, and treatment effects. Note that this generality allows for applications to open trials and trials using incentives, which is especially useful to research in economic development, public health, education, and criminology (see Technical Appendix 1 and [8]). Moreover, the possibility that *E*_*B*_ < 0 allows for the possibility of nocebo effects, and for studying their interaction with treatment.

## Data

To look for empirical evidence of interactions between treatment and behavior, we conducted a meta-analysis of antidepressant trials. Here, our approach is predicated on the two-by-two design above, in which randomization occurs twice: when assigning participants to the high- or low-probability treatment arms, and when assigning participants to the treatment or control group. This ensures that all subpopulations are comparable. While such trials have yet to be run, we can still implement estimators $\hat{T}$, $\hat{B}$, and $\hat{I}$ by using multiple trials of the same drug for which there is significant variation in the participants’ probability of treatment. As all meta-analyses suffer from possible confounds—different participant populations may differ in unobserved ways—our results should be interpreted as only suggestive evidence of the utility of two-by-two trials. Actual trials are needed for a more definitive answer.

We use data originally collected in Fournier et al., which exhaustively searched for all similar, placebo-controlled blind trials of antidepressants where participant-level data is available [9] (see Technical Appendix 2). This data is particularly appropriate for our purpose, as behavioral changes during treatment are thought to be important for depression, but complementarities between behavioral changes and treatment are not well understood. Moreover, as this was the only data we tried to obtain, and the fact that it was collected by outside authors, should reduce concerns about multiple-hypothesis testing.

Of the six trials of interest, three are of the selective serotonin re-uptake inhibitor (SSRI) paroxetine, and three are of the tricyclic antidepressant (TC) imipramine. The treatment probabilities in the SSRI trials are *p* = 50%, *p* = 65%, and *p* = 67%, and the treatment probabilities in the TC experiments are *p* = 50%, *p* = 50%, and *p* = 70% (see Technical Appendix 2 for additional details). All six trials use the Hamilton Depression Rating Scale (HDRS), which ranges from 0 to 40, with greater scores indicating more severe depression. Our health impact of interest *Y*_*τ*, *p*_ is the reduction in HDRS over the trial period. This data fits in the two-by-two trial framework by setting *p*_*L*_ = 50%, and *p*_*H*_ to encompass 65–70%. Note that the U.S. Food and Drug Administration (FDA), and human subject review committees, require the disclosure of the probability of treatment to all participants.

## Results

We first looked for evidence that participants behave in a systematically different manner when they are treated with high probability and when they are treated with low probability. While the full range of behaviors that participants engage in is not observable, all six trials report whether a participant dropped out of the experiment. For more on how to model dropout rates and their importance for inference, see [10, 11, 12, 13].

**Fig 2:**
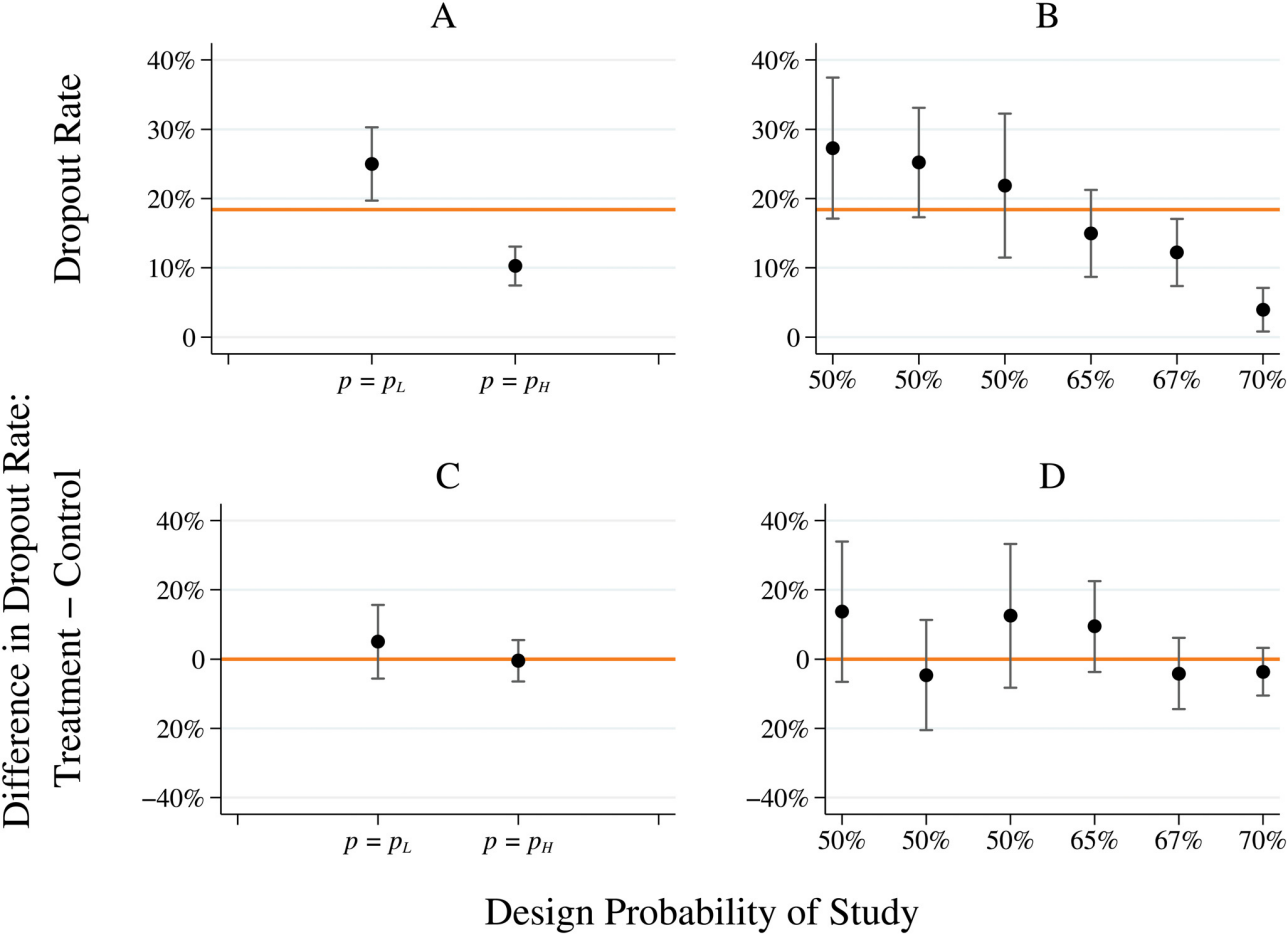
Dropout rates are significantly higher in low-probability trials. (A) and (B) show the dropout rate for low versus high treatment probability trials, and for all six individual trials, respectively. (C) and (D) show the difference in the dropout rate when comparing participants who were treated versus those that received no treatment. All graphs show point estimates (dots) and 95% confidence intervals centered at the point estimate.

Fig 2, panel (A) shows the dropout rates, with 95% confidence intervals, in the *p*_*L*_ and *p*_*H*_ trials. It is clear that the dropout rate is significantly lower in the *p*_*H*_ trials (*p*-value < 0.001). This evidence is reassuring given that the difference between *p*_*H*_ and *p*_*L*_ is moderate (*p*_*H*_ corresponds to 2/1 odds of treatment, versus 1/1 odds for *p*_*L*_).

Moreover, as shown in Fig 2, panel (B), this is true trial-by-trial. This leads to a simple statistical test. We test the alternative hypothesis that higher probabilities of treatment are associated with lower dropout rates against the null that different populations have random dropout rates, independent of treatment probabilities, and these dropout rates can be greater or less than the median dropout rate, which is equal to 18.4%. To be maximally conservative, in the null hypothesis suppose that the probability that any given population has a high or low dropout rate is 50%. Under the null hypothesis, the probability that all three low probability of treatment trials have a high dropout rate and all three high probability of treatment trials have a low dropout rate is $\frac{1}{64}<0.02$. That is, we can reject the null with *p*-value < 0.02.

Fig 2, panels (C) and (D) show that this difference in dropout rates between trials is not due to the treatment—that is, it is not due to the fact that people who are treated drop out with lower probability. In particular, in both sets of trials, and in each trial individually, there is not a statistically significant difference in the dropout rates between the treatment and control group. Indeed, in three out of the six trials the dropout rate is higher (although not statistically so) among those who are treated than among those in the control group.

**Fig 3:**
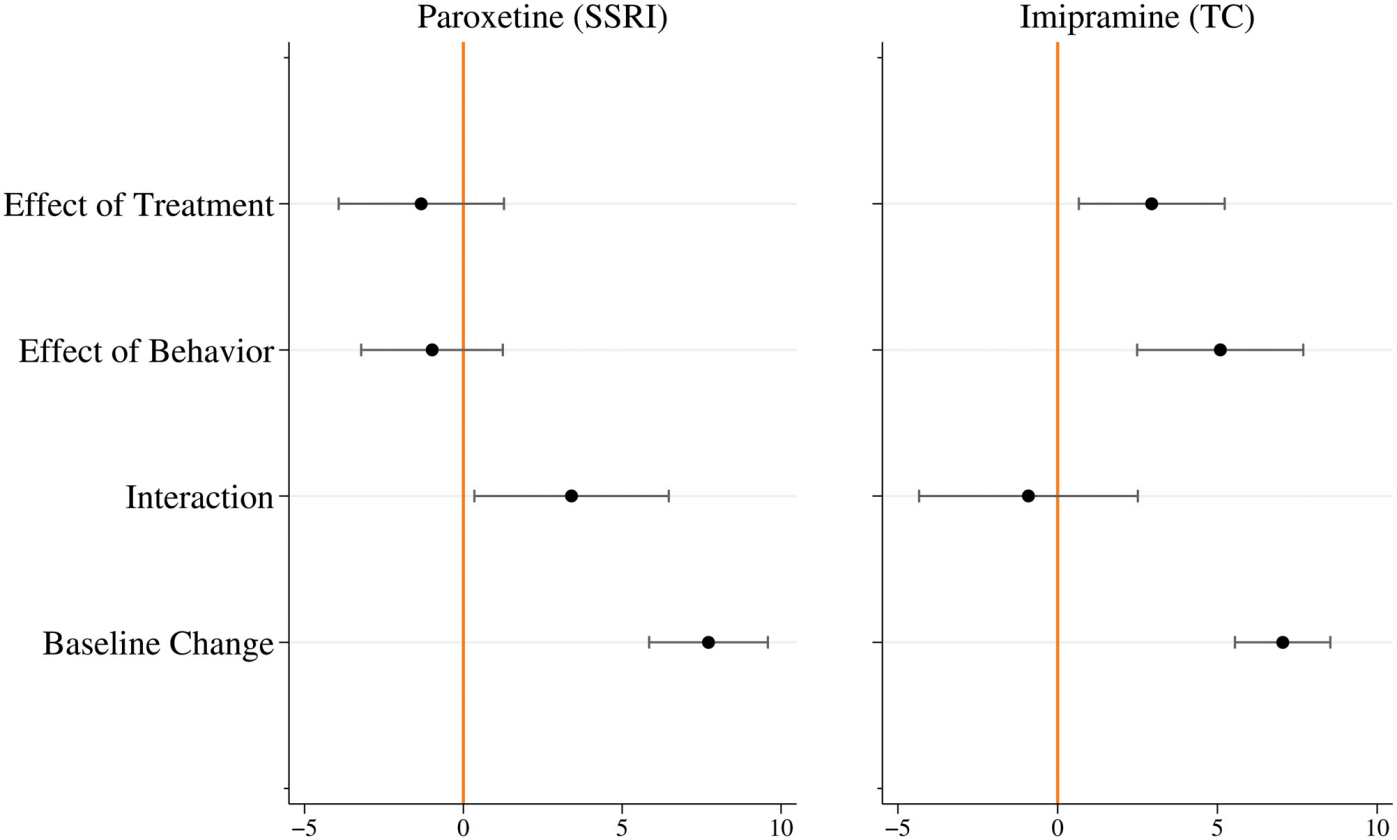
Behavior matters for both the SSRI and TC, but in different ways. The panels show the effect size (change in HDRS score) as point estimates (dots) and 95% confidence intervals centered at the point estimate constructed from hetero-skedastic consistent standard errors [14].

We next probe the specific influence of behavior on impacts (the change in the HDRS score). Fig 3 reports estimates and 95% confidence intervals for $\hat{T}$, $\hat{B}$, and $\hat{I}$. The analysis shows that both types of drugs induce large, statistically-significant behavioral effects, but that these behavioral effects are conceptually very different. In the case of the SSRI paroxetine, there is no pure effect of behavior or pure effect of treatment, but there is a strong, statistically significant, decrease in depression due to an interaction between treatment and behavior ($\hat{I}=3.41$, s.e. = 1.56, *p*-value < 0.03 two-tailed). Participants who are more confident that they are being treated change their behavior in a way that makes the drug more effective, although we do not know what behaviors are changing. This positive interaction effect cannot be obtained without the treatment and therefore should be assigned to the value added of the drug. Moreover, the existence of this interaction effect shows that further research aimed at understanding which behaviors lead to this effect is warranted. Note that without this interaction, Paroxetine appears to have no value added.

In contrast, the TC imipramine has a pure treatment effect, a pure effect of behavior ($\hat{B}=5.09$, s.e. = 1.32, *p*-value < 0.01 two-tailed), but no interaction effect between treatment and behavior. The effect of behavior alone should not be attributed to the drug. The positive behavioral effect of the TC imipramine indicates that there is a placebo effect in those studies, and that none of the studies of either drug shows evidence of a nocebo effect. However, two-by-two trials may uncover nocebo effects with other treatments.

## Discussion

In conclusion, we show that, both in theory and on the basis of available data, standard blind trials can fail to account for the full value added of a treatment when there are interaction effects between treatment and behavior. We propose the use of two-by-two blind trials, which randomize both treatment and behavior by varying the probability of treatment across different participants. This allows for separate identification of the effects of treatment, behavior, and their interaction.

There is ample scope for the existence of the interactions identified by two-by-two trials across a range of medical interventions. The potential for such interactions is determined by the nature of the placebo effect in specific conditions. Here we have highlighted behavioral changes, whereby a patient’s optimistic belief in the therapeutic benefit of a treatment may translate into a potentially observable change in their daily activity. However, placebo effects can also occur through physiological effects, the nature which is becoming increasingly well understood across a range of conditions [15]. For example, in chronic pain, placebo effects involve activation of endogenous analgesic (opioidergic) mechanisms [16]. In Parkinson’s disease, they involve activation of the dopamine system [17]. In both cases, placebos affect the molecular mechanisms targeted by pharmacological agents. There is also a growing body of evidence pointing to neurally-induced placebo-dependent modulation of inflammatory responses, likely to be clinically relevant for conditions such as psoriasis and asthma, as well as other immunological conditions [18]. Similar physiologically-mediated placebo effects are present in ulcer medicines (H_2_ blockers and PPIs) and cholesterol lowering medicines (statins) [2]. In all these cases, two-by-two trials can help evaluate plausible interaction effects between treatment and the patient’s physiological response to anticipation of treatment.

In addition, in a number of these domains, especially depression and pain, recent research has identified nocebo effects [19, 20]. These effects are often associated with clinician comments that emphasize the side-effects of treatments [21]. This suggests that our techniques could be applied to understanding the nocebo effect by randomizing the information to clinicians about treatment probability of patients (other work of ours develops the theory behind this suggestion further [8]). However, it also emphasizes that when employing the two-by-two trials here, it is important to blind clinicians not just to patients’ treatment status, but also to patients’ probability of treatment, lest they discuss side-effects more thoroughly with those with a higher probability of treatment.

Our methodology could be applied to any experiment in which a placebo is administered. While placebos are rare in evaluations outside of medicine, recent work with agricultural technologies in development economics shows this may be possible in a larger range of studies than previously thought [22]. Indeed, using our framework, this work finds significant placebo effects in evaluating new seed varieties. This suggests that our method could be fruitfully applied in field experiments in economics and public health to surmount some of the issues intrinsic to a standard randomized controlled trials in field settings [23].

We also note that interaction effects could be negative. In this case, standard blinded controlled trials might fail to identify potentially harmful interactions between behavioral or placebo effects and an intervention [24]. This could be through either the intended mechanism of the intervention, or an unwanted side-effect. Either way, our analysis raises a new mechanism by which positive trial data might not only fail to translate into real-world efficacy, but could mask deleterious effects.

It is important to note that our evidence relies on a meta-analysis of existing trials in which probability of treatment is not properly randomized. Hence our results can only be interpreted as suggestive, and proper two-by-two trials are needed to validate our results. Note, however, that the data from two-by-two trial designs can be used to identify the main effects of a treatment at little additional cost in power, even though the specific identification of interactions may itself require more participants. In other words, two-by-two designs are not any less powerful than standard designs as regards the identification of conventional differences between treatment and placebo, and will provide more accurate estimates of a treatment’s value added if significant interaction effects exist ([8] includes more discussion of sample sizes and power).

Notwithstanding our contention that new trials should follow a two-by-two design when behavioral and placebo effects are thought to be important, we also contend that, where possible, interaction effects should be incorporated into meta-analyses of existing trial data. Our results show that this can lead to different conclusions than when interaction effects are not considered.

## Technical Appendices

Technical Appendix 1 provide a formal, general interpretation of estimators $\hat{T}$, $\hat{B}$, and $\hat{I}$. Technical Appendix 2 address a potential confound in our empirical analysis.

### Technical Appendix 1 A General Model and Derivation of $\hat{T}$, $\hat{B}$, and $\hat{I}$

We now provide the general model underlying the interpretation of estimators $\hat{T}$, $\hat{B}$ and $\hat{I}$. This model allows for arbitrary heterogeneity among participants, described by participant-specific types *θ* ∈ Θ ⊂ ℝ^*n*^′^^, that summarize all observed and unobserved factors affecting a participant’s outcomes. This includes individual traits relevant for therapeutic effects, but also behavioral traits affecting a participant’s propensity to engage in various behaviors. Behavior is described by a vector *b* ∈ *B* ⊂ ℝ^*n*^. Altogether a participant’s behavior *b*_*θ*_(*p*) will depend both on type *θ* and probability of treatment *p*. In all generality, outcomes for a participant of type *θ* can be written as

$$\begin{array}{c} Y_{\tau ,p,\theta }=\mu_{\theta }\left(\tau ,b_{\theta }\left(p\right)\right)+\varepsilon_{\tau ,\theta ,p}, \end{array}$$ (2)

where *μ*_*θ*_(*τ*, *b*_*θ*_(*p*)) is the expected outcome for participants of type *θ* under treatment status *τ* and behavior *b*_*θ*_(*p*). The error term *ɛ*_*τ*, *θ*, *p*_ represents differences in outcomes due to other unobserved factors, and has expectation 𝔼[*ɛ*_*τ*, *θ*, *p*_∣*θ*] = 0.

Consider treatment probabilities *p*_*L*_ and *p*_*H*_ such that 0 < *p*_*L*_ < *p*_*H*_ < 1, this requirement being necessary for Eq (1) to be estimatable. Estimators $\hat{T}$, $\hat{B}$ and $\hat{I}$ can be written as

$$\begin{array}{ccc} \hat{T} & = & {𝔼}_{\theta }\left[\mu_{\theta }\left(1,b_{\theta }\left(p_{L}\right)\right)-\mu_{\theta }\left(0,b_{\theta }\left(p_{L}\right)\right)\right],\\ \hat{B} & = & {𝔼}_{\theta }\left[\mu_{\theta }\left(0,b_{\theta }\left(p_{H}\right)\right)-\mu_{\theta }\left(0,b_{\theta }\left(p_{L}\right)\right)\right],\\ \hat{I} & = & {𝔼}_{\theta }\left[\mu_{\theta }\left(1,b_{\theta }\left(p_{H}\right)\right)-\mu_{\theta }\left(0,b_{\theta }\left(p_{L}\right)\right)\right]-\hat{T}-\hat{B}, \end{array}$$

where randomization of both probability of treatment *p* and treatment status *τ* ensures that all expectations are taken over the same distribution of types *θ*. Hence, for *τ* ∈ {0,1} and *p* ∈ {*p*_*L*_, *p*_*H*_}, outcomes *Y*_*τ*, *p*_ can be expressed as

$$\begin{array}{c} Y_{\tau ,p}=𝔼\left[Y_{0,p_{L}}\right]+\tau \times \hat{T}+1_{p=p_{H}}\times \hat{B}+\tau \times 1_{p=p_{H}}\times \hat{I}+U_{Y} \end{array}$$ (3)

where 𝔼[*U*_*Y*_∣*p*, *τ*] = 0 because *τ* and *p* are randomly assigned. Eq (3) generalizes Eq (1), and formalizes that $\hat{T}$, $\hat{B}$, and $\hat{I}$ respectively capture the effect of treatment alone, the effect of change in behavior alone, and on effects between treatment and behavior when estimated by OLS. However, note here that behavior refers now to the distribution of behaviors among the participants.

This framework allows us to relate our contribution to that of Malani [2] and Malani and Houser [3]. These papers use similar variation in probability of treatment to identify placebo effects, and also advocate for incorporating variation in treatment probabilities into randomized trials, but do not address complementarities between treatment and behavior. In particular, in the language of this paper, the data collected by Malani [2] shows that $\hat{I}+\hat{B}>0$ for ulcer medicines (H_2_ blockers and PPIs) and cholesterol lowering medicines (statins). However, as the high-probability trials examined in that paper have a probability 1 of treatment (*p*_*H*_ = 1), it cannot separately identify $\hat{I}$ and $\hat{B}$, which is key to evaluate the value added of a treatment.

Note that this speaks to a more general issue: what should be done when treatment probabilities in a meta-analysis cannot cleanly be divided into high (*p*_*H*_) and low (*p*_*L*_)? In this case, the term **1**_*p* = *p*_*H*__ in Eq (3) can be replaced by *b*(*p*), where this is a monotonic function of the probability of treatment. While the specific form of that function should depend on the analyst’s prior about the shape of the response curve of the placebo or nocebo effect to the probability of treatment, it likely makes sense to begin the analysis with a linear function.

### Technical Appendix 2 A Potential Confound

In any meta-analysis, the fact that the participant populations in different trials may not be similar can confound the analysis (the two-by-two blind trials described in the paper would resolve this issue by randomizing both probability of treatment and treatment status). To alleviate these concerns, we provide more details about the trials analyzed in our empirical work, and investigate initial severity of depression as a potential confound. Details of the underlying trials are presented in Table 1, which is a reproduction of Table 1 in Fournier et al. [9], with the addition of a line indicating the intended probability of treatment in each trial. The original table also contained extensive information explaining decisions to include or exclude additional data from the analysis. In all cases we have followed exactly the same protocols. The interested reader can also refer to Fig 1 and surrounding text in Fournier et al. [9] for a complete description of those authors’ search search and excluding criteria.

**Table 1:** Details of 6 Studies of Medication and Placebo for Depressed Outparticipants (Table 1 of Fournier et al., 2010).

Trial:	Barrett et al.	DeRubeis et al.	Dimidijan et al.	Elkin et al.	Philipp et al.	Wichers et al.
Probability of treatment	50%	67%	65%	50%	70%	50%
Disorder	Minor^a^	MDD	MDD	MDD	MDD	MDD
No. of intake evaluations^b^	1	2	1	2	1	2
No. of treatment sites	2	2	1	3	18	8
Medication	Paroxetine	Paroxetine	Paroxetine	Imipramine	Imipramine^c^	Imipramine
Target dose, mg/d	20–40	50	50	150–250	100	100–200
Blinded Evaluations^d^	Yes	Yes	Partial	Yes	Partial	Partial
HDRS version	17-item	Modified 17-item	Modified 17-item	Modified 17-item	17-item	17-item
Minimum intake severity^e^	10	20	14	14	18	18
Sample Analyzed^f^	F–ITT	F–ITT	M–ITT	M–ITT	M–ITT	Complied with protocol
Treatment Duration, wks.^g^	11	8	8	8	8	6

Abbreviations: F–ITT, full intent to treat; HDRS, Hamilton Depression Rating Scale; MDD, major depressive disorder; Minor, minor depressive disorder; M–ITT, modified intent to treat.

^a^The Barrett et al. [25] trial also included participants diagnosed with dysthymia. These participants were not included in the current analysis.

^b^The Elkin et al. [26], DeRubeis et al. [27], and Wichers et al. [28] trials required participants to meet inclusion criteria in each of 2 consecutive evaluations that were held at least 1 week apart.

^c^The Philipp et al. [29] trial also included a *Hypericum* extract condition. Data from this condition were not included in the current analyses.

^d^“Yes” indicates that independent blind evaluators conducted evaluations of symptom severity at every assessment. “Partial” indicates that evaluations were conducted at each session by the treating pharmacotherapists. Treating phamacotherapists were blind to treatment condition.

^e^Six participants from the Elkin et al. [26] sample registered scores less than 14 on the HDRS at intake (2 from the imipramine and 4 from the placebo conditions) and 1 participant from the DeRubeis et al. [27] trial registered a score less than 20 (in the paroxetine condition). These participants were retained in the present analyses.

^f^The Barrett et al. [25] and DeRubeis et al. [27] studies used a full intent-to-treat design whereby all participants randomized to treatment were included in the analysis. The Dimidjian et al. [30], Elkin et al. [26], and Philipp et al. [29] studies used a modified intent-to-treat approach whereby data from only those participants who attended at least 1 treatment session or who had 1 postbaseline score were included. The Wichers et al. [28] trial included only those participants who met minimum compliance requirements for a protocol from a related research question (this sample did include treatment dropouts).

^g^Treatment in the Elkin et al. [26] trial was provided for 16 weeks. Because target doses were reached by the 8-week assessment, only data through week 8 were analyzed to improve comparability between the studies.

There are a few differences in the table that are worth exploring. The first is that three of the studies use a modified-intent-to-treat analysis, and thus may be dropping a large quantity of data which could affect the results. A closer look at these studies ameliorates this concern somewhat. The Dimidijan et al. study drops some data in a way that is orthogonal to assigned treatment status, and does so before any treatment has been administered. This cannot affect the results. The Elkin et al. study uses a modified-intent-to-treat design in the paper, but the data we have available to us contains all participants, so we conduct a full-intent-to-treat analysis. Finally, the Philipp et al. study drops less than 5% of the participants (12 of 263), although it is unclear whether the decision to drop data is related to treatment status. This is unlikely to affect results, although it is impossible to say with certainty. This brings up another important point about the Phillip et al. study: while the probability of being treated with Imipramine in that study (versus the placebo) is 70%, the experiment also included an arm that was treated with *Hypericum* extract, which proved to be just as effective as Imipramine. The probability of receiving any treatment (versus the placebo) was thus 85%. We use 70% in the analysis for consistency with Fournier et al., but the higher 85% probability of treatment would not change any of our results, and may explain the particularly low dropout rate in this study as shown in Fig 2.

Second, it is worth noting that although the trials are done across a wide range of time (1989, 1999, and 2008 for imipramine and 2001, 2005, and 2006 for paroxetine), they were all conducted when the treatments in question were well-established: the first study of imipramine was in 1958, and paroxetine was first marketed in 1992.

Finally, an important difference across the trials reported in Table 1, which can be addressed statistically, is that the low-probability-of-treatment paroxetine trial also had a participant population with lower initial HDRS scores. this is a potentially important confound, as more severe initial depression has been associated with larger effects of antidepressant treatment [9]. We attempt to control for initial severity by using a regression framework that includes both probability of treatment and initial severity as explanatory variables. Table 2 reports the results.

**Table 2:** Controlling for the effects of initial severity of depression in anti-depressant trials.

Dependent Variable	HDRS reduction (*Y*_*τ*, *p*_)					
	Paroxetine (SSRI)			Imipramine (TC)		
Treatment: $\hat{T}$	-1.32 (1.32)	-1.32 (1.33)	-1.32 (1.33)	2.94 (1.16)	1.97 (1.11)	1.73 (1.10)
High Probability of Treatment: $\hat{B}$	-0.98 (1.13)	-1.58 (1.17)	-1.29 (1.16)	5.09 (1.32)	5.45 (1.28)	5.17 (1.30)
High Probability × Treatment: $\hat{I}$	3.41 (1.56)	3.01 (1.59)	3.10 (1.58)	-0.91 (1.74)	-0.90 (1.64)	-0.77 (1.66)
Severe Depression (Initial HDRS ≥ 25)		2.30 (1.52)			4.48 (1.66)	
Severe Depression × Treatment		1.84 (2.08)			3.03 (2.13)	
Severe Depression (Initial HDRS ≥ 27)			2.94 (1.72)			2.67 (2.96)
Severe Depression × Treatment			3.31 (2.67)			6.00 (3.42)
Constant	7.72 (.95)	7.72 (.95)	7.72 (.95)	7.04 (.76)	5.99 (.74)	6.79 (.74)
N	384	384	384	334	334	334

Notes: Specifications estimated using OLS with hetero-skedastic consistent standard errors in parenthesis [14].

Table 2 replicates our previous empirical results in the first and fourth column, and includes controls for the initial severity of depression for each participant in the second, third, fifth and sixth columns. Following [9], we examine two cutoffs for severe depression: an initial HDRS ≥ 25 and an initial HDRS ≥ 27. Regardless of the cutoff, the results are qualitatively unchanged across different specifications.

For the SSRI paroxetine, the interaction effect $\hat{I}$ maintains significance at traditional levels, but the coefficient attenuates slightly ($\hat{I}=3.01$, s.e. = 1.59, *p*-value < 0.06 two tailed when using an initial HDRS ≥ 25, and $\hat{I}=3.10$, s.e. = 1.58, *p*-value < 0.05 two tailed when using an initial HDRS ≥ 27). The results for the TC imipramine are virtually unchanged by the inclusion of controls for severe depression. This is not surprising as the three TC trials themselves, and the participants therein, are very similar.

As a further robustness check we produce results for dropout rates similar to those of Fig 2. To control for treatment status explicitly, we model the process of dropping out as

$$\begin{array}{c} D_{\tau ,p}=\beta_{0}+\beta_{1}\times \tau +\beta_{2}\times 1_{p=p_{H}}+U_{D}, \end{array}$$ (4)

where **1**_*p* = *p*_*H*__ takes a value of one if *p* = *p*_*H*_, and zero otherwise. The parameter of interest here is *β*_2_, which corresponds to the change in dropout rates for high probability of treatment trials.

The first and third columns of Table 3 show the same result as in Fig 2. A higher probability of treatment is associated with a statistically significant decrease in the probability of dropping out of the trial (*β*_2_ = −0.16, s.e. = 0.056, *p*-value < 0.01 two tailed). In contrast, treatment status itself is statistically unrelated to the decision to dropout. The second, third, fifth and sixth columns show that there is no qualitative effect of including controls for initial severe depression.

**Table 3:** Controlling for the effects of initial severity of depression in anti-depressant trials.

Dependent Variable	Dropout (*D*_*τ*, *p*_)					
	Paroxetine (SSRI)			Imipramine (TC)		
Treatment: *β*_1_	0.041 (.039)	0.043 (.039)	0.042 (.039)	-0.0073 (.039)	-0.0030 (.039)	-0.0044 (.039)
High Probability of Treatment: *β*_2_	-0.15 (.047)	-0.16 (.049)	-0.15 (.047)	-0.20 (.039)	-0.20 (.039)	-0.20 (.039)
Severe Depression (Initial HDRS ≥ 25)		0.071 (.049)			-0.090 (.045)	
Severe Depression (Initial HDRS ≥ 27)			0.067 (.069)			-0.040 (0.57)
Constant *β*_0_	0.25 (.046)	0.25 (.046)	0.25 (.046)	0.24 (.032)	0.26 (.033)	0.25 (.032)
N	384	384	384	334	334	334

Notes: Specifications estimated using OLS with hetero-skedastic consistent standard errors in parenthesis [14].

In summary, our empirical findings are robust to controls for initial severity of depression. However, it is important to note that the very fact that the probability of treatment varies at a level of aggregation higher than the individual can lead to issues with estimating standard errors [31]. Standard fixes for this issue, such as clustering of standard errors, or the inclusion of random effects, are known to produce incorrect results with a small number of observations (six, in this case). Indeed, this is the case here: using either of these fixes results in t-statistics in excess of 10 on the coefficients of interest. Thus, we continue to emphasize that while this evidence is reassuring, it is only suggestive. Proper two-by-two trials are needed to validate our results.
